# Effects of *Nigella sativa* on Performance, Blood Profiles, and Antibody Titer against Newcastle Disease in Broilers

**DOI:** 10.1155/2021/2070375

**Published:** 2021-06-14

**Authors:** Alireza Talebi, Masoud Maham, Siamak Asri-Rezaei, Pouya Pournaghi, Mohammad-Sadegh Khorrami, Amir Derakhshan

**Affiliations:** ^1^Department of Poultry Health & Diseases, Faculty of Veterinary Medicine, Urmia University, P.O. Box 57153-1177tbox57153-1177, Urmia, Iran; ^2^Department of Large Animal Medicine, Faculty of Veterinary Medicine, Urmia University, P.O. Box 57153-1177, Urmia, Iran; ^3^Graduated of Veterinary College, Urmia University, Urmia, Iran; ^4^Department of Biology, Payame Noor University, Tehran, Iran

## Abstract

Recent anxiety about resistance to chemical drugs has elevated the position of phytogenic feed additives including *Nigella sativa* in preventive strategy in the poultry industry. During this study, a completely randomized experiment was designed to investigate the efficacy of different levels (0 to 16%) of *N. sativa* seeds supplemented in the diet of broilers on performance, immune responses, and hematological and biochemical parameters. The results indicated the following: (a) Supplementation of 1% *N. sativa* seeds in diet had the highest positive effects and 16% *N. sativa* had the highest significant (*p*=0.03) adverse effects on weight gain, while up to 2% *N. sativa* seeds in the diet reduced feed conversion ratio (FCR) whereas 4% and over that increased the FCR. (b) Chickens fed with a diet containing 1% *N. sativa* seeds had the highest antibody titers, but those fed with 16% *N. sativa* seeds had the lowest antibody titers at end of the experiment. (c) Dietary inclusion of *N. sativa* seeds increased hemogram parameters and the group fed with 16% *N. sativa* seeds had the highest values on day 21 until the end of the experiment. (d) Supplementation of *N. sativa* seeds decreased in WBC and lymphocytes but increased heterophils, H/L, monocytes, eosinophils, and basophils percentages. Supplementation of up to 2% of *N. sativa* seeds in broiler's diets elaborated serum level of those parameters, while supplementation of ≥ 4% *N. sativa* seeds decreased their serum levels. In conclusion, supplementation of *N. sativa* seed (1-2%) in broiler diets, as a multipurpose natural growth promoter, improves performance, elevates humoral immune responses, affects serum biochemical profiles of broiler chickens, and induces changes in their hemogram and leukogram, while there are no side, residual, and hazardous effects.

## 1. Introduction

Phytogenic feed additives “phytobiotic” are assuming a position of prime importance in the poultry industry [1–3] and could be used in feed or water [4]. Among the promising phytogenic immune-stimulants, *Nigella sativa* (*N*. *sativa*) is a miracle medicinal herb having a long history with a rich religious background [5, 6]. *N*. *sativa* seeds are used for the treatment of some disorders and illnesses including fever, common cold, headache, asthma, various Gram-positive and Gram-negative microbial infections [7], aflatoxicosis [8], antiethanol hepatotoxicity [9], and alleviation of adverse effects of heat stress [10], as well as to expel worms from the intestines. The possible biochemical active components of *N*. *sativa* and their pharmacological effects have been investigated [11, 12], and their pharmacological actions as immune stimulation, antioxidant, anti-inflammatory, antimicrobial, and antiparasitic activities[5, 13–17] have also been studied. Recently, the potential effects of *N*. *sativa* seeds' bioactive compounds (such as thymoquinone, *α*-hederin, and nigellidine) in control of the COVID-19 pandemic have also been documented [18–20]. In the production of healthy food, supplementation of *N*. *sativa* seeds to poultry diets could be recommended as a natural growth promoter instead of antibiotics. Previous reports indicate that diets containing different levels of *N*. *sativa* seeds improve the performance and carcass characteristics in broilers [5, 21]. The inclusion of *N*. *sativa* seeds in layers' diet also increases egg production as well as eggs' quality [22] and serum triglycerides in layers [23]. However, *N*. *sativa* also affects blood profile and biochemical parameters [24, 25] and prevents atherogenesis by decreasing LDL-cholesterol and increasing HDL cholesterol [26]. *N*. *sativa* can control infectious diseases and regulate adaptive immunity via enhancement of macrophages [27]. Effects of *N*. *sativa* on cell-mediated immunity [28] and humoral immunity [29] have also been investigated. Although it has been documented that *N*. *sativa* has immunomodulatory effects [5], the type of immune responses against various pathogens provoked by *N*. *sativa* may need further investigations. However, interest in herbal medicines and their multiple beneficial utilization in poultry health and performance has been recently reviewed [5, 6, 30–32]. Finding the best beneficial and safe phytogenic feed additives as the most suitable substitute to antibiotic promoters is much more today's research topic [1, 2, 4, 33–35].

The objective of this research was a comprehensive study on the efficacy of *N*. *sativa* as a multipurpose feed additive on performance, blood profiles, and humoral immune responses in broiler chickens. To the best of our knowledge, the closest reports to our study in the literature are works of Al-Beitawi et al. [29], Durrani et al. [36], Shewita and Taha [37], Ghasemi et al. [38], Hossain et al. [39], Shirzadegan et al. [40], Singh and Kumar [41], and Aydogan et al. [42], and the present study has a lot of differences with them including the use of various percentages of *N*. *sativa* seeds in diets and weekly measurement of performance, immune responses, serum biochemical parameters, hemogram, and leukogram indices.

## 2. Materials and Methods

### 2.1. Ethics of Experimentation

All experimental procedures were carried out according to the standard animal experimentation protocols of the Veterinary Ethics Committee of Faculty of Veterinary Medicine, Urmia University (Ref. IR-UU-AEC-3/902/DA).

### 2.2. Experimental Design and Chicken Management

In a completely randomized experiment, one hundred sixty unsexed one-day-old chicks (Ross-308) were divided into 8 treatment groups (20 chicks/group) with 4 replicates. The treatment groups were T0 = control 1 (without vaccination and without supplementation of *N. sativa* in diet), T1 = control 2 (T0 + Vaccination), T2 = T1 + *N. sativa* 0.5%, T3 = T1 + *N. sativa*1%, T4 = T1 + *N. sativa* 2%, T5 = T1 + *N. sativa* 4%, T6 = T1 + *N. sativa* 8%, and T7 = T1 + *N. sativa* 16%. After leg labeling, the chicks were housed in separated boxes, and environmental conditions (ambient temperature, humidity, lighting, ventilation, nutrition, etc.) are provided according to the technical instructions of Ross-308 for the broiler management guide. The basal diets (Table 1) were prepared according to the Ross-308 broiler nutritional specification guide. Energy and protein of *N. sativa* seed treated (T2–T7) groups' diets were roughly balanced by reducing 0.1 Kg oil + 0.47 Kg corn + 0.43 Kg soybean meal for supplementation of each 1 Kg of *N. sativa* seed.

### 2.3. Vaccines and Vaccination Routes

According to break-through level 1:8 (log 2^−3^) of maternal antibody [43], live ND clone 30 vaccine (eye-drop route) together with ND oil (subcutaneous route) is used for primary vaccination on day 9 of age, and the chickens were revaccinated with live ND clone-30 vaccine by eye-drop method [44] on the 21st day.

### 2.4. Sampling Procedures

Blood samples for hematological tests (using EDTA anticoagulant-treated syringes) and blood samples for evaluation of immunity and biochemical tests were collected on day one and then at weekly intervals until the end of the experimental period (42 days old), as previously described [45]. Blood samples were labeled according to the number and date of sampling and kept at room temperature until clotted, and then, the sera were separated for serological tests.

### 2.5. Evaluation of Performance

The performance of the chickens for each week was determined.

### 2.6. Evaluation of Antibody Titer

On day one and day seven of age, serum samples were used to evaluate maternally derived antibodies of the chicks to determine the best time of first vaccination. On day 14 and weekly interval, serum samples were used to assess protective immune response derived from vaccination against ND by application of hemagglutination inhibition (HI) test as it has been reported that the HI test is an excellent indicator of the immune status and disease resistance of a flock, especially to assess protective responses following vaccination because, unlike ELISA, HI test correlates well with the more laborious virus neutralization (VN) assays [44].

### 2.7. Hematological Tests

Hematological parameters such as red blood cell (RBC) counts, PCV, hemoglobin (Hb) concentration, white blood cells (WBC) counts, and percentages of heterophils, lymphocytes, monocytes, eosinophils, basophils, and heterophils to lymphocytes (**H**/**L**) ratio were determined by routine methods as previously described [46].

### 2.8. Biochemical Tests

Serum biochemical parameters values were determined by using an autoanalyzer (Technicon RA 1000 TM, Hartwell, LA, USA) and specific Pars-Azmun kits (Pars-Azmun Co., Tehran, Iran) as follows: albumin (ALB) concentration (mg/dL with the bromcresol green method at 546 nm WL), alkaline phosphatase (ALP) concentration (U/L with DGKC method at 405 nm WL), alanine transaminase (ALT), or serum glutamate-pyruvate transaminase (SGPT) concentration (U/L with Opt. standard method, IFCC at 340 nm WL), aspartate transaminase (AST) or serum glutamic oxaloacetic transaminase (SGOT) concentration (U/L with Opt. standard method, IFCC at 340 nm WL), calcium concentration (mg/dL with cresolphthalein complex method at 550–590 nm WL), cholesterol concentration (mg/dl with chod-pap method at 546 nm WL), glucose concentration (mg/dL with god-pap method at 500–546 nm WL), phosphorus concentration (mg/dL with UV method at 340 nm WL), total protein concentration (mg/dL with Biuret method at 540–546 nm WL), and triglyceride concentration (mg/dL with gpo-pap enzymatic method at 546 nm WL).

### 2.9. Statistical Analysis

The results were analyzed by using the SPSS software (Version 21; SPSS Inc., Chicago, USA). The means for the treatments showing significant differences in the ANOVA were compared using the Post Hoc Tukey and Wilcoxon (nonparametric tests, 2 related samples) tests used for analyzing data of each parameter within the group and between groups. The relationship between age and parameter contents was ascertained through the Spearman correlation coefficient test. Differences were considered significant at *p* < 0.05.

## 3. Results

### 3.1. Performance

**Figure 1:**
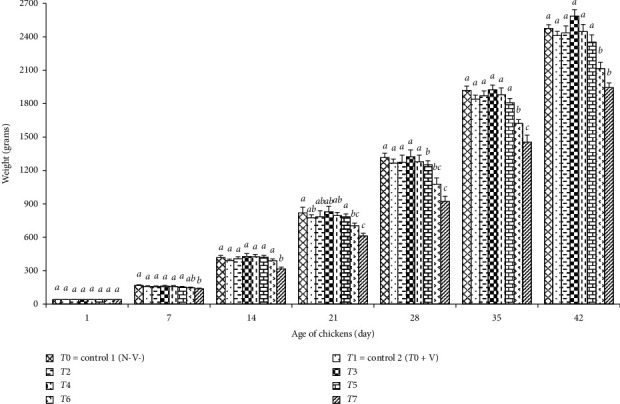
Effects of *N*. *sativa* on the weight of broilers: T0 = control 1 (without vaccination and without supplementation of *N*. *sativa* in diet), T1 = control 2 (T0 + vaccination), T2 = T1 + *N*. *sativa* 0.5%, T3 = T1+*N*. *sativa* 1%, T4 = T1 + *N*. *sativa* 2%, T5 = T1 + *N*. *sativa* 4%, T6 = T1 + *N*. *sativa* 8%, and T7 = T1 + *N*. *sativa* 16%. Different alphabetical letters indicate significant (*p* < 0.05) differences between the means of the groups.

*N. sativa* had some effects on the performance of the chickens (Table 2), and as shown in Figure 1, the chickens of group T3 fed with a diet containing 1% *N. sativa* seeds had the highest weight at the end of the experiment but differences with those of the groups (T0–T5) were not significant (*p* > 0.05), but those with those of the groups (T6 and T7) were significant (*p* < 0.05). Dietary inclusion of 16% *N. sativa* seeds had a significant (*p* < 0.03) depressive effect on weight gain of chickens (T7) during the experimental period. As shown in Table 2, the feed efficiency ratio was affected by the level of *N. sativa* seeds, and supplementation (T3 and T4) of the diet with 1%–2% *N. sativa* seeds had decreased FCR but 8%–16% increased FCR.

### 3.2. Immune Response

**Figure 2:**
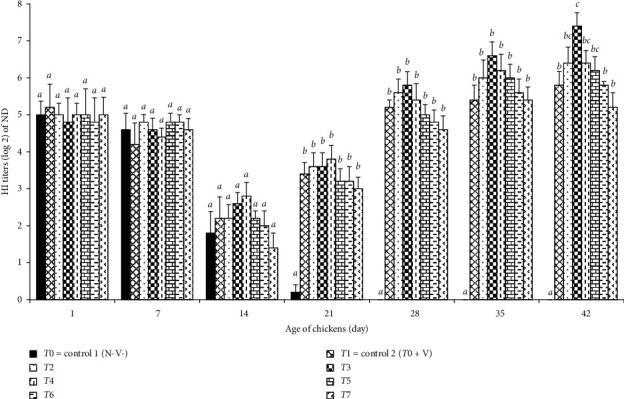
Effects of *N*. *sativa* on ND antibody titer of broilers: T0 = control 1 (without vaccination and without supplementation of *N*. *sativa* in diet), T1 = control 2 (T0 + vaccination), T2 = T1 +*N*. *sativa* 0.5%, T3 = T1 + *N*. *sativa* 1%, T4 = T1 + *N*. *sativa* 2%, T5 = T1 + *N*. *sativa* 4%, T6 = T1 + *N*. *sativa* 8%, and T7 = T1 + *N*. *sativa* 16%. Different alphabetical letters indicate significant (*p* < 0.05) differences between the means of the groups.

As shown in Figure 2, maternal antibody titer (MDA) of the chickens against ND decreased gradually and reached the points that are considered as negative in the control group T0 (environmental contamination control) on day 21 of age, and the comparison of average antibody titers of the groups during 2 weeks of age could be explained where maternal antibody of the chick's yolk may contribute to MDA of chicks during 3 days of age, and reduction of the MDA starts on day 4 of age based on half-life time (4-5 days) in case of ND [42]. On the contrary, antibody titers of ND of the vaccinated chickens (group T1–T7) were increased after vaccination at the 14th day of the experiment. Different levels of *N*. *sativa* seeds in diet affected antibody titers against ND in the vaccinated groups and the chickens (group T3) fed with 1% *N*. *sativa* supplementation had the highest level of antibody titers, while chickens of group T7 (16% *N*. *sativa* seed in diet) had the lowest level of antibody titers on days 28, 35, and 42 of age (Figure 2). Weekly comparison of antibody titers of the groups revealed the following: (1) Differences among the groups were not significant (*p* < 0.05) on days 1, 7, and 14 of age, indicating that vaccination and *N*. *sativa* supplementation in feed did not strongly affect the MDA reduction rate of the chickens. (2) A significant difference (*p* < 0.05) between the unvaccinated and vaccinated groups on day 21 of age to the end of the experiment may indicate the effectiveness of the vaccination, while nearly undetectable antibody titers of unvaccinated chickens may confirm that there was no environmental or cross-contamination during the experimental period. (3) The lack of significant differences among the vaccinated groups on days 21, 28, and 35 of age may indicate that supplementation of different levels of *N*. *sativa* in diet affected the immune responses but did not have a strong effect on antibody titers of the treated groups, at least in a short period. Comparison antibody titers of the vaccinated groups at the end (day 42) of the experiment showed that antibody titers of group T3 (vaccinated and feed with a diet containing 1% *N*. *sativa* seeds) differed significantly (*p*=0.027) from that of group T1 (vaccinated but feed without *N*. *sativa* seeds diet) and from those of group T6 (vaccinated and feed with diet containing 8% *N*. *sativa* seeds) and group T7 (vaccinated and feed with the diet containing 16% *N*. *sativa* seeds) significantly (*p*=0.02 and *p*=0.01, respectively) indicating effectiveness of different levels of *N*. *sativa* seeds supplementation in diets, as dietary inclusion of up to 1% *N*. *sativa* seeds had the best immunomodulatory effects on the level of antibody titer following vaccination.

### 3.3. Hemogram

As shown in Table 3, RBC and PCV as well as Hb values are age-dependent, and their values increased as the chickens get older. Weekly comparison of these parameters showed that supplementation of *N. sativa* seeds in the diet increases the values of RBC, PCV, and Hb, and the group of chickens fed with 16% supplemented *N. sativa* seeds had the highest values and significantly (*p* < 0.05) differed from those of control groups (T0 and T1) as well as those of the treated groups (T2-T4) indicating that increasing effects of *N. sativa* seeds on hemogram parameters are dose-dependent (Table 3).

### 3.4. Leukogram

As shown in Table 4, WBC count of the vaccinated control 2 group (T1 = V + N−) was higher (*p* > 0.05) than that of the control 1 group (T0 = V−N−) from day 14 up to the end of the experiment indicating that WBC counts were affected by ND vaccination. Supplementation of *N*. *sativa* seeds affects leukogram parameters by decreasing WBC counts and lymphocytes and increasing heterophils, *H*/*L* ratio, monocytes, eosinophils, and basophils percentages from day 21 of age until the end of the experiment. Weekly comparison (Table 4) of the groups indicates that the group (T7) fed with 16% *N*. *sativa* seeds had the lowest WBC counts and lymphocyte percentages and their differences with control 2 group (T1 = T0 + V) were significant (*p*=0.013 and *p*=0.008, respectively). But, values for *H*/*L* ratio and percentages of heterophils, monocytes, eosinophils, and basophils of the group fed (T7) with 16% *N*. *sativa* seeds were the highest and their values differed from those of the control 2 group (T1 = T0 + V) significantly (*p*=0.001).

### 3.5. Biochemical Parameters

#### 3.5.1. Albumin and Total Protein

The concentration of albumin and total protein of control chickens increased during 6 weeks of the experimental period indicating that these parameters are age-dependent. A weekly comparison of the groups (Table 5) showed that supplementation of up to 2% of *N*. *sativa* seeds in the diet increases serum level of these parameters whereas 4% and over that decrease concentrations of these elements in the serum of broiler chickens. Overall, albumin and total protein levels of chickens fed with a diet containing different *N*. *sativa* seeds were higher than those of the control groups (T0-T1) and the differences were significant (*p* < 0.05) when compared with those of the control groups (T0-T1).

#### 3.5.2. Glucose

As shown in Table 5, weekly comparison of the control groups (T0-T1) revealed that the serum glucose concentration of the chickens is decreased by age, and vaccination against ND did not affect its level. A weekly comparison of the treated groups indicated that the effects of *N*. *sativa* on serum glucose were dose-dependent. On the other hand, increasing the effect on serum glucose of the chickens was observed by 0.5 to 2% of *N*. *sativa* seed supplementation in diet, while serum glucose of the chickens fed with 4% and over that decreased gradually (Table 5). Overall, the chickens fed with different concentrations of *N*. *sativa* seeds in the feed had higher glucose level than those of the control groups (T0-T1), and a weekly comparison of the serum glucose level of the groups showed that on age 14 up to the end of the experiment, chickens of the group (T4) fed with 2% *N*. *sativa* diet had the highest level of serum glucose and differences between the groups were significant (*p* < 0.05).

#### 3.5.3. Calcium and Phosphorus

As shown in Table 5, supplementation of up to 2% of *N*. *sativa* seeds in diet had increasing effects on levels of serum calcium and phosphorus, while 4% and over that had decreasing effects. Chickens fed with 2% of *N*. *sativa* seeds in the diet had the highest level of serum calcium and phosphorus that significantly (*p* < 0.05) differed from those of the control groups (T0 and T1).

#### 3.5.4. Cholesterol and Triglyceride

As shown in Table 6, high levels of cholesterol and triglyceride due to yolk residue reduced sharply in the 1^st^ week, and then their levels reduced gradually during the experimental period, indicating that their concentrations are age-dependent at least in broilers up to 42 days of age. Weekly comparison of *N*. *sativa* treated and control groups showed that inclusion of *N*. *sativa* seeds in broiler's diets reduces the concentration of both these parameters, and chickens of group T7 fed with 16% *N*. *sativa* seeds had the lowest level and differed significantly (*p* < 0.05) from those of the control groups (T0-T1).

#### 3.5.5. Serum Enzymes (ALP, ALT, and AST)

As shown in Table 6, the lack of significant differences between group T0 and group T1 indicates that vaccination does not affect the serum enzyme levels, and levels of both ALP and AST were decreased by age, while those of ALT were increased. Weekly comparison (Table 6) of the groups revealed the following. (a) The levels of ALP decreased by the inclusion of *N. sativa* seeds in the diet during the experimental period and group T7 had the lowest levels of ALP which significantly (*p* < 0.05) differed from those of control groups (T0-T1) from age 14 to 42 days. (b) Inclusion of up to 2% of *N. sativa* seeds in the diet increased the levels of ALT while 8% and over that decreased levels of ALT. Therefore, group T4 (2% *N. sativa*) had the highest level of ALT and group T7 (16% *N. sativa*) had the lowest level of ALT. The mean differences between the control groups with groups T4 (2% *N. sativa*) and T7 (16% *N. sativa*) were significant (*p* < 0.05) on days 28, 35, and 42 of age). The level of AST is decreased gradually during the experimental period. Weekly comparison (Table 6) of the groups revealed that the decreasing effect of *N. sativa* on the AST level is dose-dependent and group T7 had the lowest levels during the whole experimental period. As shown in Table 6, the mean differences of the groups T6 (8%) and T7 (16%) significantly (*p* < 0.05) differed from those of the rest groups on day 21of age to the end of the experimental period.

## 4. Discussion

### 4.1. Performance

Comparison of the vaccinated group (T1) with the unvaccinated group (T0) indicated that vaccination had a little (*p* > 0.05) adverse effect on weight gains of the chickens as expected due to vaccination stress. As shown in Figure 1, our results about the efficacy of *N. sativa* supplementation on the performance of broiler chickens are in agreement with the results obtained by Miraghaee et al. [24], Ghasemi et al. [38], Hossain et al. [39], and Ali et al. [47] who reported that 1% *N. sativa* seeds in diet improved the performance of broiler chickens but there are some differences between our results with those obtained by Shewita and Taha [37] who observed a higher weight in chickens fed with 2% *N. sativa* seed supplementation diet when compared with the weight of those that received 10% of *N. sativa* seeds in their diet, or Durrani et al. [36] who reported a higher weight gain in 40 g/kg (4%) of *N. sativa* seeds in the diet. Overall, most studies indicate that a higher percentage of *N. sativa* seeds in the diet may reduce feed intake and finally affects the average weight of broiler chickens as observed in this study (Table 2) for groups T6 (8% N. sativa seeds) and T7 (16% *N. sativa* seeds), and the mean of the latest differed significantly (*p* < 0.05) from those of the control (T0-T1) groups (Figure 1).

### 4.2. Immune Response

The lack of an increase in antibody titer of chickens of the unvaccinated group (T0) during the experimental period confirmed that no environmental or cross-contamination has occurred. Lack of significant differences among treated groups during days 1–14 of age indicates that supplementation of *N. sativa* seeds on diet had no significant effects on MDA reduction of the chickens. A significant (*p*=0.04) difference between antibody titers of group T3 and group T1 indicates that dietary inclusion of 1% *N. sativa* seeds had the highest immunomodulatory effects, while that of group T7 (16% *N. sativa* seeds) had immunosuppressive effects. The results observed during this study (Figure 2) indicate that *N. sativa* is able to promote antibody response (mostly IgG) in chickens as previously reported [5, 17, 29, 36, 47, 48]. Higher antibody titers (log 2^–7.4^) of chickens (group T3) observed during this study are in the range of expected titers that could be induced via vaccinations by two live plus one killed vaccines [49, 50]. Our results about the efficacy of different levels of *N. sativa* seeds in the diet on immune responses of chickens also is in harmony with the results obtained by Hossain et al. [39] who reported that supplementation of 1% *N. sativa* seeds in a broiler diet improved the development of immunity but is in disagreement with the results obtained by Khan et al. [51]. However, our observation on antibody titers is in disagreement with those of Shewita and Taha [37] and Ghasemi et al. [38] who reported that 20 g/Kg of *N. sativa* had the highest immunomodulatory effects, and this inconsistency could be attributed to the lack of 1% concentration in their work. Our observation on a decrease in lymphocytes percentage together with an increase in antibody titers following vaccination against Newcastle diseases is controversial to the results obtained by Islam et al. [52] who reported a decrease in antibody titer together with a rise in peripheral lymphocytes with an explanation that the numbers of antigen-specific-antibody secreting B-cells were less. As recent reports indicated that *N. sativa* has induced a significant increase in antibody titers against Newcastle disease (ND) and infectious bursal disease (IBD) in broilers [29, 36], therefore, the reasons for this controversy results may be due to the nature of pathogenic agents, type of treatments (vaccination versus challenge), kind of experimental animal, different components and dosage of *N. sativa*, and the role of macrophages in the activation of Th2 cells and their roles in the activation of B-cells for further differentiation into plasma cells. For example, the increase in antibody titer against avian infectious bronchitis (IB) has been observed by Durrani et al. [36] but has not been observed by Al-Beitawi et al. [29] or various dosages of *N. sativa* had different effects on antibody titers against different pathogenic agents (ND, IBD, and IB) in same experimental animals “broiler chickens” [36].

### 4.3. Hemogram

As shown in Table 3, the lack of significant (*p* > 0.05) differences between hemogram values of the control 1 (T0) and those of control 2 (T1) groups indicates that vaccination alone did not significantly influence the hemograms. The results (Table 3) obtained during this study in regard to values of some hematological parameters (RBC, PCV, and Hb) in broiler chickens are in agreement with those previously reported [53].

### 4.4. Leukogram

Our results (Table 4) on the reduction effects of *N*. *sativa* on leukocyte counts of the chickens are in agreement with previous reports [54, 55]. The results obtained during this study revealed that supplementation of *N*. *sativa* seeds in broiler's diet increased the H/L ratio (Table 4), and our results are in disagreement with the results obtained by Ali et al. [26] who reported that supplementation of 1% *N*. *sativa* may reduce the heterophil/lymphocyte (H/L) ratio. An increase in eosinophil values observed during this study (Table 4) is in line with the results obtained by Ahmad et al. [13] who reported that *N*. *sativa* rises peripheral blood lymphocytes and eosinophils in Long-Evans rats. It has been documented that monocytes-macrophages play an indispensable role in the immune system [55] and an increase in their numbers may indicate the immunomodulatory effects of *N*. *sativa*.

### 4.5. Biochemical Parameters

In general, biochemical parameters of poultry may be influenced by various factors and the results of this study on biochemical parameters (Table 5) were compared with reported reference ranges [40–42, 56–58].

#### 4.5.1. Albumin and Total Protein

As shown in Table 5, alternation on concentrations of albumin and total protein of the chickens fed with *N*. *sativa* seeds observed during this study have also been reported by Miraghaee et al. [24]. However, weekly comparison (Table 5) values of total protein and albumin of the treated groups showed that differences among the groups were not significant (*p* > 0.05) as previously reported [42, 59] but in disagreement with the results observed by Singh and Kumar [41]. As shown in Table 5, increasing of protein and albumin concentrations of the chickens fed with 0.5–2% and decreasing of those chickens fed with 4–16% *N*. *sativa* may indicate that the efficacy of *N*. *sativa* on protein and albumin concentrations is dose-dependent.

#### 4.5.2. Glucose

The results obtained during this study (Table 5) on the level of serum glucose of chickens are in agreement with recent reports [29, 40] but in disagreement with other reports [41, 42]. Some controversial observations could be explained where the type of the extract, whole seed, and duration of consumption may exert different effects on glucose level as has been observed by Mohtashami [60] who reported that *N*. *sativa* did not significantly affect the fasting blood glucose level but significantly decreased its levels during oral glucose tolerance test (OGTT) when compared to diabetic control by affecting the time course of glucose absorption from the intestine.

#### 4.5.3. Calcium and Phosphorus

The results obtained for calcium and phosphorus during this study (Table 5) are in agreement with previous reports that supplementation of *N*. *sativa* in the feed of broiler diets significantly increases the level of serum calcium [61,62] because *N*. *sativa* possesses estrogenic activity in regulating calcium-regulating hormones [61].

#### 4.5.4. Cholesterol and Triglyceride

Because of yolk sac in day-old chicks, the concentration of both cholesterol and triglyceride were very high at the beginning of the experiment, but as shown in Table 6, by aging, their values reduced, and their levels in the control groups at age 42 days were in the range as previously reported [63]. The results obtained during this study on the serum cholesterol and triglyceride levels of broiler chickens are in agreement with previous reports that *N*. *sativa* reduces serum cholesterols of chickens [21, 24, 29, 37–40, 47], in particular HDL cholesterol [26]. The reduction in serum cholesterol and triglycerides has been attributed to the lowering effect of black cumin (thymoquinone and monounsaturated fatty acids) on the synthesis of cholesterol by hepatocytes or fractional reabsorption from the small intestine [29]. It is also believed that phytosterols of *N*. *sativa* inhibit cholesterol absorption and may interfere with the reabsorption of endogenous cholesterol [26]. Moreover, it has also been reported that *N*. *sativa* decreases plasma concentrations of cholesterol by stimulating bile acid excretion [5], and a reduction in plasma cholesterol level could also be attributed to the effects of black cumin on the cholesterol excretion into the intestine [21, 26]. As shown in Table 6, the lowering effects of *N*. *sativa* supplementation in the diet of commercial layers on cholesterol could be useful because lower egg yolk cholesterol of commercial layers' eggs is highly desirable for human consumption [5], but it may not be useful in breeders because of egg yolk role in embryo development.

#### 4.5.5. Serum Enzymes

Serum enzymes including ALP, ALT, and AST are mainly monitored for the evaluation of liver damage [3,9], and antioxidant activity of *N*. *sativa* components such as thymoquinone may play a vital role in the prevention of liver tissue [64]. The reduction of ALP and AST levels of broiler chickens fed with *N*. *sativa* supplementation observed during this study (Table 6) is in agreement with previous reports [21, 40, 59] but is in disagreement with that observed by Shewita and Taha [37] who reported that serum glutamic pyruvic transaminase (SGPT) level significantly increased with *N*. *sativa* supplementation. As shown in Table 6, increasing effects of *N*. *sativa* seeds on levels of ALT observed during this study have also been reported [40] and may indicate positive effects of *N*. *sativa* on liver health because ALT level is commonly used clinically as a biomarker for liver health.

## 5. Conclusion

Supplementation of *N*. *sativa* seed (1-2%) in broiler diets, as a multipurpose natural growth promoter, improves performance, elevates humoral immune responses, affects serum biochemical profiles of broiler chickens, and induces changes in their hemogram (Hb, PCV) and leukogram, while there are no side, residual, and hazardous effects.

## Figures and Tables

**Table 1:** Ingredients and nutrients of the basal diet.

Ingredients (E kcal, CP %)	Starter (1–10 days)	Grower (11–10 day 25 days)	Finisher >26 days)
Corn (3350, 8.9)	51%	42%	36%
Soya meal (2300, 44)	42%	35.5%	32.4%
Wheat (2900, 12)	—	10%	14%
Barley (2600, 11)	—	4%	8%
Oil (9000, 0)	3.3%	5.4%	7%
Oyster shell	1.6%	1.4%	1.2%
Monocalcium phosphate	1.3%	1.2%	1.1%
Vitamin premix	0.25%	0.25%	0.25%
Mineral premix	0.25%	0.25%	0.25%
Cl Na	0.15%	0.15%	0.15%
DKAB complex	0.1%	0.05%	0.05%
L-Lysin	0.1%	0.08%	0.08%
DL-Methionin	0.1%	0.1%	0.1%
N. sativa seed (3450, 23)	0	0	0
Total	100.15	100.33	100.58
Energy and protein of 1 kg *N. sativa* seeds were simply considered equal to nutrients of 0.47 kg corn + 0.43 kg soya meal + 0.1 kg oil; therefore, for each 1 kg of *N. sativa* seeds supplementation, 0.47 kg corn + 0.43 kg soya meal + 0.1 kg oil were reduced.			
Calculate major nutrients			
Energy (E) kcal/KgF	3001.5	3103.5	3202.8
Protein (CP) %	23.02	21.0	20.02
E/CP	130.35	147.66	160.14
Calcium %	0.973	0.865	0.802
Phosphorus %	0.448	0.44	0.40

**Table 2:** Effects of *N. sativa* on weekly performance (mean ± SEM) of broilers.

Age (day)	Group	Weight (g)	Weight gain (g)	Feed (g/week)	FCR (weekly)	FCR (total)
Day 1	T0–T7	39.64 ± 0.08NS	—	—	—	—
Day 7	T0	167.5 ± 5.6a	128.0 ± 5.4a	205 ± 5.5	1.60 ± 0.07a	1.22 ± 0.04a
	T1	157.8 ± 6.5a	118.6 ± 4.6b	195 ± 4.6	1.65 ± 0.05 b	1.23 ± 0.05a
	T2	157.1 ± 5.8a	117.4 ± 4.7bc	190 ± 4.0	1.62 ± 0.03ab	1.21 ± 0.03a
	T3	160.4 ± 6.2a	120.8 ± 5.2 b	193 ± 3.5	1.60 ± 0.04a	1.20 ± 0.04a
	T4	157.8 ± 7.5a	118.0 ± 4.6bc	189 ± 4.0	1.60 ± 0.05a	1.20 ± 0.05a
	T5	151.3 ± 6.0a	111.7 ± 5.0c	181 ± 3.7	1.62 ± 0.06ab	1.20 ± 0.06a
	T6	146.0 ± 3.5ab	106.3 ± 6.2dc	175 ± 4.0	1.65 ± 0.05b	1.20 ± 0.04a
	T7	137.0 ± 3.4b	97.3 ± 4.0e	175 ± 4.5	1.80 ± 0.06c	1.27 ± 0.05a
Day 14	T0	417.8 ± 19.2a	250.3 ± 11.0a	451 ± 15.0	1.80 ± 0.07a	1.57 ± 0.06a
	T1	391.7 ± 11.9a	234.0 ± 12.5bd	440 ± 16.5	1.88 ± 0.06ab	1.62 ± 0.05ac
	T2	406.3 ± 19.1a	249.2 ± 13.0ca	461 ± 20.0	1.85 ± 0.06ab	1.60 ± 0.06ac
	T3	426.0 ± 26.9a	265.6 ± 12.8c	483 ± 19.0	1.82 ± 0.07ab	1.58 ± 0.07ba
	T4	424.8 ± 21.3a	267.0 ± 13.2c	480 ± 17.5	1.80 ± 0.06a	1.58 ± 0.06ba
	T5	420.0 ± 16.3a	268.7 ± 14.0c	483 ± 18.0	1.80 ± 0.08a	1.59 ± 0.05ac
	T6	388.4 ± 16.4a	242.4 ± 12.3da	460 ± 19.0	1.90 ± 0.07bc	1.63 ± 0.04ac
	T7	316.0 ± 16.1b	179.0 ± 11.0e	349 ± 16.5	1.95 ± 0.08c	1.66 ± 0.05c
Day 21	T0	819.0 ± 50.0a	401.2 ± 21.0a	674 ± 32.0	1.68 ± 0.06ab	1.62 ± 0.04a
	T1	774.7 ± 27.0ab	383.0 ± 19.5 b	648 ± 29.0	1.69 ± 0.07ab	1.65 ± 0.06ab
	T2	784.1 ± 54.0ab	377.8 ± 17.5 b	627 ± 28.0	1.66 ± 0.05a	1.63 ± 0.05a
	T3	828.8 ± 70.0a	402.8 ± 18.0a	665 ± 27.5	1.65 ± 0.06a	1.61 ± 0.04a
	T4	794.1 ± 28.0ab	369.5 ± 16.9c	609 ± 25.0	1.65 ± 0.04a	1.61 ± 0.06a
	T5	783.5 ± 26.0ab	363.0 ± 16.4c	607 ± 24.5	1.67 ± 0.06ab	1.62 ± 0.04a
	T6	703.5 ± 24.0bc	315.1 ± 15.0d	541 ± 26.8	1.74 ± 0.06bc	1.68 ± 0.05ab
	T7	611.9 ± 24.0c	295.9 ± 13.8e	523 ± 24.5	1.77 ± 0.08c	1.71 ± 0.07 b
Day 28	T0	1315.0 ± 40a	496.0 ± 24.0a	808 ± 26.0	1.63 ± 0.05ab	1.62 ± 0.06ab
	T1	1266.4 ± 36a	491.7 ± 24.8a	807 ± 27.0	1.64 ± 0.05ab	1.64 ± 0.07ab
	T2	1274.4 ± 64a	490.3 ± 26.0a	800 ± 25.0	1.63 ± 0.06ab	1.63 ± 0.04ab
	T3	1322.6 ± 61a	493.8 ± 25.5a	780 ± 24.5	1.58 ± 0.05b	1.60 ± 0.06a
	T4	1277.5 ± 60a	483.4 ± 24.0a	778 ± 22.6	1.60 ± 0.07ab	1.60 ± 0.07a
	T5	1250.4 ± 37b	466.9 ± 24.3b	779 ± 24.0	1.67 ± 0.06ab	1.64 ± 0.05ab
	T6	1076.4 ± 57bc	372.9 ± 23.5c	624 ± 26.0	1.68 ± 0.07ca	1.68 ± 0.06b
	T7	921.0 ± 47c	309.1 ± 22.5d	572 ± 25.0	1.85 ± 0.08d	1.75 ± 0.07c
Day 35	T0	1919.0 ± 39a	604.0 ± 35.0a	1056 ± 33.0	1.70 ± 0.07a	1.64 ± 0.05a
	T1	1840.6 ± 36a	574.2 ± 29.5ab	993 ± 29.0	1.73 ± 0.07a	1.67 ± 0.05ab
	T2	1871.0 ± 44a	596.6 ± 26.0a	1032 ± 35.0	1.73 ± 0.06a	1.66 ± 0.06ab
	T3	1925.0 ± 41a	602.4 ± 28.0a	1036 ± 32.0	1.72 ± 0.05a	1.64 ± 0.04a
	T4	1880.0 ± 61a	602.5 ± 32.0a	1043 ± 38.5	1.73 ± 0.07a	1.66 ± 0.05ab
	T5	1808.0 ± 39a	557.6 ± 28.5bc	1003 ± 35.0	1.80 ± 0.08b	1.69 ± 0.07ab
	T6	1623.0 ± 34b	548.6 ± 27.0bc	999 ± 38.0	1.82 ± 0.07bc	1.72 ± 0.07b
	T7	1454.6 ± 63c	533.6 ± 25.0c	997 ± 28.0	1.87 ± 0.08c	1.80 ± 0.08c
Day 42	T0	2476.2 ± 33a	557.2 ± 28.0a	1131 ± 35.0	2.03 ± 0.09a	1.73 ± 0.07a
	T1	2412.1 ± 37a	571.5 ± 26.5a	1171 ± 36.0	2.05 ± 0.08a	1.76 ± 0.08a
	T2	2436.0 ± 62a	565.0 ± 24.0a	1164 ± 38.0	2.06 ± 0.09a	1.75 ± 0.08a
	T3	2586.0 ± 57a	661.0 ± 29.5b	1348 ± 41.0	2.04 ± 0.07a	1.74 ± 0.07a
	T4	2447.6 ± 64a	567.5 ± 26.5a	1191 ± 37.0	2.10 ± 0.08ab	1.75 ± 0.07a
	T5	2352.8 ± 64a	544.8 ± 26.0a	1183 ± 33.0	2.17 ± 0.09b	1.79 ± 0.06ab
	T6	2115.1 ± 57b	492.1 ± 24.5c	1082 ± 36.0	2.20 ± 0.09b	1.84 ± 0.05bc
	T7	1944.2 ± 42b	489.6 ± 26.0c	1082 ± 37.0	2.21 ± 0.08b	1.90 ± 0.08c

*Note*. T0 = control 1 (without vaccination and without supplementation of *N. sativa* in diet), T1 = control 2 (T0+vaccination), T2 = T1 + *N. sativa* 0.5%, T3 = T1 + *N. sativa* 1%, T4 = T1 + *N. sativa* 2%, T5 = T1 + *N. sativa* 4%, T6 = T1 + *N. sativa* 8%, and T7 = T1 + *N. sativa* 16%. Different superscript letters in each column/week indicate significant (*p* < 0.05) differences between groups, and NS stands for not significant (*p* > 0.05).

**Table 3:** Effects of *N. sativa* on hemogram (mean ± SEM) of broiler chickens.

Age (day)	Group	RBC (×106)	PCV (%)	Hb (g/dl)
Day1	T0−T7	4.34 ± 0.029NS	24.2 ± 0.00NS	9.68 ± 0.047NS
Day 7	T0	5.00 ± 0.054a	27.2 ± 0.00a	10.98 ± 0.015a
	T1	5.44 ± 0.024a	31.0 ± 0.00a	11.40 ± 0.044a
	T2	5.5 ± 0.024a	31.8 ± 0.00a	11.64 ± 0.024a
	T3	5.54 ± 0.024a	31.6 ± 0.00a	11.60 ± 0.044a
	T4	5.52 ± 0.040a	30.6 ± 0.00a	11.20 ± 0.063a
	T5	5.50 ± 0.019a	29.8 ± 0.00a	11.08 ± 0.051a
	T6	5.46 ± 0.015a	29.3 ± 0.00a	10.92 ± 0.020a
	T7	5.42 ± 0.048a	29.0 ± 0.00a	10.7 8 ± 0.020a
Day 14	T0	4.32 ± 0.048a	26.6 ± 0.00a	8.28 ± 0.074a
	T1	4.34 ± 0.060a	26.8 ± 0.00ab	8.36 ± 0.021ab
	T2	4.44 ± 0.024a	27.4 ± 0.00a	8.56 ± 0.067ab
	T3	4.48 ± 0.020abc	27.8 ± 0.00b	8.70 ± 0.080b
	T4	4.50 ± 0.067b	27.8 ± 0.00b	8.72 ± 0.019b
	T5	4.54 ± 0.064bcd	28.0 ± 0.00bc	8.76 ± 0.015bc
	T6	4.62 ± 0.073cb	28.6 ± 0.00cbd	8.96 ± 0.018bc
	T7	4.76c ± 0.024d	29.6 ± 0.01d	9.28 ± 0.073d
Day 21	T0	4.62 ± 0.048a	28.6 ± 0.00a	8.98 ± 0.073a
	T1	4.64 ± 0.060a	28.8 ± 0.00a	9.04 ± 0.019ab
	T2	4.74 ± 0.024ab	29.4 ± 0.00ba	9.22 ± 0.073a
	T3	4.78 ± 0.020ac	29.8 ± 0.00b	9.34 ± 0.060bcde
	T4	4.80 ± 0.067bcd	29.8 ± 0.00bc	9.34 ± 0.018c
	T5	4.84 ± 0.087bc	30.0 ± 0.00bcd	9.4 ± 0.016bcd
	T6	4.94 ± 0.048cb	30.6 ± 0.00cbd	9.58 ± 0.014dce
	T7	5.12 ± 0.031d	31.6 ± 0.00d	9.88 ± 0.073e
Day 28	T0	4.78 ± 0.024a	29.6 ± 0.00a	9.28 ± 0.073a
	T1	4.80 ± 0.054ab	29.8 ± 0.00ab	9.30 ± 0.008ab
	T2	4.88 ± 0.048ab	30.4 ± 0.00abc	9.52 ± 0.073abc
	T3	4.96 ± 0.040ac	30.8 ± 0.00bc	9.64 ± 0.060bc
	T4	4.98 ± 0.059bc	30.8 ± 0.00cd	9.64 ± 0.009cd
	T5	5.00 ± 0.001bc	31.0 ± 0.00bcd	9.7 ± 0.007bcd
	T6	5.10 ± 0.057cd	31.8 ± 0.00dce	9.88 ± 0.008dce
	T7	5.26 ± 0.024db	32.6 ± 0.00edc	10.18 ± 0.073edc
Day 35	T0	5.12 ± 0.048a	31.6 ± 0.00a	9.88 ± 0.073a
	T1	5.14 ± 0.060a	31.8 ± 0.00ab	9.94 ± 0.000ab
	T2	5.24 ± 0.024 ab	32.4 ± 0.00abc	10.12 ± 0.073ab
	T3	5.28 ± 0.020 abc	32.8 ± 0.00bcd	10.24 ± 0.060bcd
	T4	5.30 ± 0.067bc	33.0 ± 0.00cd	10.24 ± 0.008c
	T5	5.34 ± 0.074bcd	33.0 ± 0.00cbd	10.30 ± 0.007c
	T6	5.42 ± 0.073cd	33.6 ± 0.00dc	10.48 ± 0.009dce
	T7	5.56 ± 0.024dc	34.6 ± 0.00ed	10.78 ± 0.073ed
Day 42	T0	5.56 ± 0.024a	34.6 ± 0.01a	10.75 ± 0.07a
	T1	5.60 ± 0.054abc	34.8 ± 0.01ab	10.86 ± 0.01abc
	T2	5.68 ± 0.048ab	35.4 ± 0.01ab	11.06 ± 0.09abc
	T3	5.76 ± 0.040abcd	35.8 ± 0.01bcd	11.20 ± 0.08bc
	T4	5.78 ± 0.080bc	35.8 ± 0.01bc	11.22 ± 0.01bc
	T5	5.80 ± 0.010bc	36.0 ± 0.01bc	11.26 ± 0.01bcd
	T6	5.90 ± 0.080cd	36.6 ± 0.01cd	11.46 ± 0.01bcd
	T7	6.06 ± 0.024d	37.6 ± 0.01dc	11.78 ± 0.07d

*Note*. RBCs: red blood cells, PCV: packed cell volume, and Hb: hemoglobin. T0 = control 1 (without vaccination and without supplementation of *N. sativa* in diet), T1 = control 2 (T0 + vaccination), T2 = T1 + *N. sativa* 0.5%, T3 = T1 + *N. sativa* 1%, T4 = T1 + *N. sativa* 2%, T5 = T1 + *N. sativa* 4%, T6 = T1 + *N. sativa* 8%, and T7 = T1 + *N. sativa* 16%. Different superscript letters in each column/week indicate significant (*p* < 0.05) differences between groups, and NS stands for not significant (*p* > 0.05).

**Table 4:** Effects of *N. sativa* on leukogram (mean ± SEM) of broiler chickens.

Age	Group	WBC (×103)	Het (%)	Lym (%)	Het/Lymp	Mon (%)	Eos (%)	Bas (%)
Day1	T0-T7	20.37 ± 0.18NS	37 ± 7NS	51.4 ± 7NS	0.94 ± 0NS	4 ± 0NS	6 ± 0NS	1.62 ± 0NS
Day 7	T0	29.1 ± 0.10a	30.0 ± 1a	55.0 ± 0.8a	0.54 ± 0.00a	4.8 ± 0.0acd	4.2 ± 0ac	6.02 ± 0a
	T1	29.2 ± 0.20a	38.8 ± 1b	51.0 ± 1b	0.76 ± 0.00b	3.4 ± 0.0 b	3.22 ± 0b	3.62 ± 0b
	T2	28.6 ± 0.21a	37.2 ± 1b	51.4 ± 0.5ab	0.72 ± 0.02b	4.0 ± 0.0ab	3.42 ± 0ab	4.02 ± 0b
	T3	26.8 ± 0.12b	34.6 ± 1ba	52.8 ± 0.7ab	0.65 ± 0.02 b	4.2 ± 0.0abc	4.02 ± 0abc	4.42 ± 0bc
	T4	25.0 ± 0.41b	31.2 ± 3ca	54.8 ± 0.0a	0.56 ± 0.03a	4.6 ± 0.0abc	4.42 ± 0c	5.02 ± 1cd
	T5	24.2 ± 0.53b	27.2 ± 1cad	56.6 ± 1ca	0.48 ± 0.09a	5.0 ± 0.0c	4.62 ± 0 cd	5.62 ± 0da
	T6	23.0 ± 0.10 cd	23.0 ± 2d	60.2 ± 1dc	0.38 ± 0.01c	5.4 ± 0.0 d	5.62 ± 0de	5.82 ± 1da
	T7	21.0 ± 0.10 d	21.2 ± 4d	60.6 ± 2d	0.34 ± 0.00c	6.4 ± 0.0e	6.02 ± 0e	5.82 ± 0da
Day 14	T0	20.72 ± 0.48ab	47.0 ± 1a	46.0 ± 0.1a	1.02 ± 0.02a	3.7 ± 0a	2.2 ± 0a	1.62 ± 0a
	T1	21.40 ± 0.50 a	45.8 ± 1a	45.8 ± 0.3a	1.00 ± 0.04a	3.8 ± 0a	2.82 ± 0a	1.82 ± 0a
	T2	19.36 ± 0.72ab	43.2 ± 1ab	46.6 ± 0.5a	0.93 ± 0.02abc	4.4 ± 0ab	3.42 ± 0ab	2.42 ± 0ab
	T3	18.82 ± 0.86bc	42.0 ± 1ba	46.6 ± 0.7a	0.89 ± 0.02b	4.82 ± 0bc	3.82 ± 0bc	2.82 ± 0bc
	T4	18.43 ± 1.56bc	41.8 ± 3bc	46.6 ± 0.0a	0.89 ± 0.04bc	4.92 ± 0bc	3.82 ± 0bc	2.92 ± 0bc
	T5	17.57 ± 0.74c	41.4 ± 1bc	46.6 ± 0.6a	0.88 ± 0.06bc	5.02 ± 0bc	4.02 ± 0bc	3.02 ± 0bc
	T6	17.18 ± 1.24c	39.0 ± 2cd	47.2 ± 0.5b	0.82 ± 0.05cd	5.62 ± 0cd	4.62 ± 0 cd	3.62 ± 0 cd
	T7	16.90 ± 0.58c	34.6 ± 4d	48.6 ± 0.8b	0.71 ± 0.02d	6.62 ± 0d	5.62 ± 0d	4.62 ± 0d
Day 21	T0	22.70 ± 0.04ab	33.6 ± 0a	52.6 ± 0a	0.64 ± 0.01a	4.62 ± 0a	6.62 ± 0a	2.62 ± 0a
	T1	22.95 ± 0.05a	33.8 ± 0a	51.8 ± 1a	0.65 ± 0.02a	4.82 ± 0ab	6.82 ± 0ab	2.82 ± 0ab
	T2	20.91 ± 0.07abc	34.4 ± 0ab	49.4 ± 0ab	0.69 ± 0.02a	5.42 ± 0abc	7.42 ± 0abc	3.42 ± 0abc
	T3	20.37 ± 0.08bcd	34.6 ± 0bd	48.6 ± 0b	0.71 ± 0.01ab	5.62 ± 0bc	7.62 ± 0b	3.62 ± 0bc
	T4	20.12 ± 0.10cbd	34.8 ± 0bd	47.8 ± 1bd	0.73 ± 0.03ab	5.82 ± 0cd	7.82 ± 0cd	3.82 ± 0cd
	T5	19.98 ± 0.07cbd	35.0 ± 0cb	47.0 ± 2cb	0.74 ± .04ab	6.02 ± 0cd	8.02 ± 0cd	4.02 ± 0cd
	T6	18.73 ± 0.12cd	35.6 ± 0dc	44.6 ± 2dce	0.80 ± 0.04bc	6.62 ± 0d	8.62 ± 0d	4.62 ± 0d
	T7	18.45 ± .05d	36.6 ± 0e	40.6 ± 0e	0.90 ± 0.02c	7.6 ± 0 e	9.62 ± 0e	5.62 ± 0e
Day 28	T0	23.80 ± 0.04a	37.6 ± 0a	45.6 ± 0a	0.82 ± 0.02a	5.62 ± 0a	7.62 ± 0a	3.62 ± 0a
	T1	24.50 ± 0.05ab	37.8 ± 0a	44.8 ± 1a	0.84 ± 0.03a	5.72 ± 0ab	7.92 ± 0a	3.82 ± 0a
	T2	22.46 ± 0.07abc	38.4 ± 0ab	42.4 ± 0ab	0.90 ± 0.02ab	6.42 ± 0abc	8.32 ± 0ab	4.52 ± 0ab
	T3	21.90 ± 0.08abc	38.5 ± 0ab	41.6 ± 0b	0.92 ± 0.02ab	6.62 ± 0bcd	8.52 ± 0ab	4.82 ± 0bc
	T4	21.67 ± 0.01ac	38.8 ± 0bc	40.6 ± 2b	0.95 ± 0.05ab	6.82 ± 0cd	8.92 ± 0bc	4.92 ± 0bc
	T5	21.53 ± 0.07c	39.0 ± 0bc	40.0 ± 2b	0.97 ± 0.06ab	7.02 ± 0dcd	9.02 ± 0bc	5.02 ± 0bc
	T6	20.28 ± 0.12dc	39.6 ± 0c	37.6 ± 2cd	1.05 ± 0.07bc	7.52 ± 0d	9.62 ± 0 cd	5.72 ± 0c
	T7	20.00 ± 0.05dc	40.6 ± 0d	33.6 ± 0d	1.20 ± 0.04c	8.52 ± 0e	10.42 ± 0d	6.92 ± 0d
Day 35	T0	25.57 ± 0.04ab	35.4 ± 0a	57.8 ± 0a	0.61 ± 0.01a	3.62 ± 0a	2.52 ± 0a	0.72 ± 0a
	T1	26.25 ± 0.05a	35.8 ± 0ab	56.8 ± 1ab	0.63 ± 0.02ab	3.92 ± 0ab	2.72 ± 0ab	0.82 ± 0ab
	T2	24.21 ± 0.07 abc	36.3 ± 0ab	54.5 ± 0abc	0.66 ± 0.01ab	4.32 ± 0abc	3.52 ± 0abc	1.42 ± 0abc
	T3	23.67 ± 0.08abc	36.5 ± 0bc	53.8 ± 0bc	0.68 ± 0.01ab	4.62 ± 0bc	3.52 ± 0bc	1.62 ± 0abc
	T4	23.42 ± 0.10bc	36.8 ± 0c	52.7 ± 1c	0.70 ± 0.03abc	4.82 ± 0bcd	3.92 ± 0cd	1.82 ± 0cd
	T5	23.28 ± 0.07bc	37.0 ± 0c	52.0 ± 2c	0.71 ± 0.04abc	5.02 ± 0cd	4.02 ± 0cd	2.02 ± 0cd
	T6	22.03 ± 0.12c	37.6 ± 0d	49.7 ± 2d	0.76 ± 0.04bc	5.52 ± 0d	4.62 ± 0d	2.62 ± 0de
	T7	21.72 ± 0.05c	38.6 ± 0e	45.7 ± 0e	0.84 ± 0.02c	6.62 ± 0e	5.62 ± 0e	3.52 ± 0e
Day 42	T0	27.12 ± 0.04ab	33.6± ± 0a	54.7 ± 0a	0.61 ± 0.01a	3.62 ± 0a	5.52 ± 0a	2.62 ± 0a
	T1	27.80 ± 0.05a	33.8± ± 0ab	53.9 ± 1ab	0.63 ± 0.02ab	3.82 ± 0ab	5.72 ± 0ab	2.82 ± 0ab
	T2	25.76 ± 0.07abc	34.4± ± 0abc	51.5 ± 0abc	0.66 ± 0.01ab	4.32 ± 0abc	6.42 ± 0abc	3.42 ± 0abc
	T3	25.22 ± 0.08abc	34.6 ± 0bc	50.6 ± 0bc	0.68 ± 0.01abc	4.62 ± 0bcd	6.52 ± 0bc	3.72 ± 0bcd
	T4	24.83 ± 0.10bc	34.9 ± 0cd	49.7 ± 1bcd	0.70 ± 0.03abc	4.82 ± 0cd	6.82 ± 0cd	3.82 ± 0cd
	T5	24.67 ± 0.07bc	35.0 ± 0cd	49.0 ± 2cd	0.71 ± 0.04bc	5.02 ± 0cd	7.02 ± 0cd	4.02 ± 0cd
	T6	23.58 ± 0.12c	35.6 ± 0d	46.7 ± 2d	0.76 ± 0.04c	5.52 ± 0d	7.62 ± 0d	4.62 ± 0d
	T7	23.30 ± 0.05c	36.6 ± 0e	42.5 ± 0e	0.86 ± 0.02d	6.62 ± 0e	8.72 ± 0e	5.62 ± 0e

*Note*. WBC: white blood cells, Het: heterophils, Lym: lymphocytes, H/L: heterophils to lymphocytes ratio, Mon: monocytes, Eos: eosinophils, and Bas: basophils. T0 = control 1 (without vaccination and without supplementation of *N. sativa* in diet), T1 = control 2 (T0 + vaccination), T2 = T1 + *N. sativa* 0.5%, T3 = T1 + *N. sativa* 1%, T4 = T1 + *N. sativa* 2%, T5 = T1 + *N. sativa* 4%, T6 = T1 + *N. sativa* 8%, and T7 = T1 + *N. sativa* 16%. Different superscript letters in each column/week indicate significant (*p* < 0.05) differences between groups, and NS stands for not significant (*p* > 0.05).

**Table 5:** Effects of *N. sativa* on biochemical parameters (Mean ± SEM) of broilers.

Age (day)	Group	Alb (g/dL)	TP (g/dL)	Glu (mg/dL)	Cal (mg/dL)	Phos (mg/dL)
Day1	T0–T7	0.62 ± 0.03NS	1.73 ± 0.10NS	276 ± 7.5NS	8.19 ± 0.07NS	2.09 ± 0.02NS
Day 7	T0	0.74 ± 0.05a	1.85 ± 0.10a	220.6 ± 304ad	8.0 ± 0.03a	3.03 ± 0.04a
	T1	0.72 ± 0.02a	1.86 ± 0.02a	216.0 ± 0.94a	8.06 ± 0.05a	30.8 ± 0.12a
	T2	0.76 ± 0.02a	1.87 ± 0.03ab	225.0 ± 2.56abd	8.26 ± 0.04a	3.52 ± 0.06b
	T3	0.78 ± 0.03a	2.06 ± 0.06b	232.8 ± 1.99bc	8.36 ± 0.11b	3.66 ± 0.08bc
	T4	0.84 ± 0.03a	2.06 ± 0.10b	240.4 ± 0.52b	9.60 ± 0.05c	3.78 ± 0.08c
	T5	0.80 ± 0.05a	1.96 ± 0.06 b	230.4 ± 2.88cd	8.18 ± 0.03ab	3.74 ± 0.04cb
	T6	0.70 ± 0.01a	1.78 ± 0.05ab	227.0 ± 1.86db	7.52 ± 0.04ad	3.71 ± 0.09cb
	T7	0.68 ± 0.03a	1.74 ± 0.08a	221.4 ± 2.04ad	7.25 ± 0.05d	3.61 ± 0.05cb
Day 14	T0	0.74 ± 0.07a	2.14 ± 0.05a	221.8 ± 1.93ad	8.50 ± 0.14a	3.26 ± 0.08a
	T1	0.68 ± 0.07a	1.92 ± 0.08abc	217.6 ± 2.18a	8.48 ± 0.16a	3.14 ± 0.03a
	T2	0.70 ± 0.05a	1.96 ± 0.15abc	239.4 ± 1.24b	8.90 ± 0.06a	3.40 ± 0.06b
	T3	0.72 ± 0.05a	2.04 ± 0.12abc	246.8 ± 3.39bc	9.06 ± 0.04a	3.48 ± 0.05b
	T4	0.80 ± 0.0a	2.26 ± 0.14a	254.6 ± 1.03c	9.06 ± 0.08b	3.86 ± 0.07c
	T5	0.70 ± 0.07a	1.90 ± 0.14abc	250.0 ± 3.56c	7.44 ± 0.12c	3.50 ± 0.06b
	T6	0.68 ± 0.06a	1.86 ± 0.08ab	244.2 ± 3.42bc	7.24 ± 0.16c	3.02 ± 0.05a
	T7	0.64 ± 0.04a	1.68 ± 0.13cb	227.4 ± 3.50d	6.94 ± 0.13c	3.10 ± 0.04a
Day 21	T0	0.94 ± 0.07a	2.34 ± 0.08a	220.8 ± 1.9ad	8.24 ± 0.16a	3.30 ± 0.07a
	T1	0.72 ± 0.07a	2.08 ± 0.10abc	224.2 ± 2.1ad	8.18 ± 0.16b	3.20 ± 0.06a
	T2	0.94 ± 0.05a	2.12 ± 0.17abc	251.2 ± 1.2b	9.50 ± 0.06b	3.66 ± 0.03 b
	T3	0.98 ± 0.05a	2.22 ± 0.12abc	269.0 ± 2.3c	9.66 ± 0.04b	3.80 ± 0.02c
	T4	1.0 ± 0.05a	2.46 ± 0.16a	276.6 ± 1.0c	10.98 ± 0.08c	3.92 ± 0.03c
	T5	0.90 ± 0.07a	2.06 ± 0.15abc	272.0 ± 2.5c	7.96 ± 0.14a	3.62 ± 0.05b
	T6	0.88 ± 0.06a	1.98 ± 0.10 b	269.2 ± 2.4c	7.76 ± 0.16a	3.14 ± 0.08ad
	T7	0.88 ± 0.04a	1.80 ± 0.15cb	224.4 ± 3.5d	7.47 ± 0.13a	3.00 ± 0.05 d
Day 28	T0	1.34 ± 0.07a	2.53 ± 0.10a	209.8 ± 1.9a	8.44 ± 0.16a	3.38 ± 0.05a
	T1	1.34 ± 0.07a	2.56 ± 0.10a	213.6 ± 2.1a	8.36 ± 0.16a	3.30 ± 0.08a
	T2	1.60 ± 0.05b	3.08 ± 0.10bcd	260.2 ± 1.2b	9.80 ± 0.08b	3.70 ± 0.03b
	T3	1.62 ± 0.05b	3.18 ± 0.09bcd	278.0 ± 2.3c	9.96 ± 0.04b	3.90 ± 0.02c
	T4	1.80 ± 0.05c	3.40 ± 0.10b	285.6 ± 1.0c	11.08 ± 0.08c	4.02 ± 0.03c
	T5	1.68 ± 0.07bc	3.18 ± 0.10bcd	281.0 ± 2.6c	8.26 ± 0.14ad	3.72 ± 0.05b
	T6	1.68 ± 0.06bc	3.04 ± 0.10cd	278.2 ± 2.4c	8.06 ± 0.16ad	3.24 ± 0.08ad
	T7	1.58 ± 0.04b	3.00 ± 0.08dc	228.0 ± 2.5d	7.76 ± 0.13d	3.10 ± 0.05d
Day 35	T0	1.54 ± 0.07a	2.94 ± 0.12a	203.8 ± 1.93a	8.54 ± 0.16a	3.40 ± 0.07a
	T1	1.54 ± 0.07a	2.94 ± 0.12a	211.6 ± 2.18a	8.68 ± 0.16a	3.32 ± 0.06a
	T2	1.70 ± 0.05ab	3.24 ± 0.11abc	255.2 ± 1.24b	9.90 ± 0.06bc	3.76 ± 0.03b
	T3	1.82 ± 0.06b	3.36 ± 0.11bc	273.0 ± 3.39c	10.05 ± 0.04bc	4.00 ± 0.02c
	T4	1.88 ± 0.05b	3.56 ± 0.10b	280.6 ± 1.02c	10.38 ± 0.08c	4.04 ± 0.04c
	T5	1.78 ± 0.07bc	3.44 ± 0.12bc	272.0 ± 3.56c	8.36 ± 0.14a	3.74 ± 0.05b
	T6	1.72 ± 0.06ab	3.22 ± 0.11abc	259.2 ± 3.42db	8.16 ± 0.16ad	3.26 ± 0.05ad
	T7	1.68 ± 0.06ac	3.18 ± 0.11ca	204.4 ± 3.50ea	7.80 ± 0.14d	3.14 ± 0.06d
Day 42	T0	1.74 ± 0.07a	3.24 ± 0.12a	180.8 ± 1.92a	8.92 ± 0.1a	3.60 ± 0.08a
	T1	1.72 ± 0.07a	3.22 ± 0.12a	194.6 ± 2.17b	9.14 ± 0.2a	3.52 ± 0.06a
	T2	1.92 ± 0.06abc	3.54 ± 0.11abc	232.2 ± 3.38c	11.68 ± 0.1b	3.96 ± 0.04b
	T3	2.02 ± 0.05bc	3.66 ± 0.10bc	250.0 ± 1.12d	11.86 ± 0.1b	4.20 ± 0.03c
	T4	2.08 ± 0.06 b	3.86 ± 0.12b	257.6 ± 3.56d	12.06 ± 0.1b	4.24 ± 0.04c
	T5	1.98 ± 0.07bc	3.74 ± 0.11bc	243.6 ± 3.42d	9.92 ± 0.2c	3.94 ± 0.05 b
	T6	1.90 ± 0.06abc	3.52 ± 0.11abc	238.2 ± 2.50c	9.72 ± 0.1c	3.46 ± 0.06a
	T7	1.85 ± 0.05ac	3.48 ± 0.11ac	198.0 ± 1.12b	9.36 ± 0.1da	3.34 ± 0.06da

*Note*. Alb: albumin, TP: total protein, Glu: glucose, Cal: calcium, and Ph: phosphorous. T0 = control 1 (without vaccination and without supplementation of *N. sativa* in diet), T1 = control 2 (T0 + vaccination), T2 = T1 + *N. sativa* 0.5%, T3 = T1 + *N. sativa* 1%, T4 = T1 + *N. sativa* 2%, T5 = T1 + *N. sativa* 4%, T6 = T1 + *N. sativa* 8%, and T7 = T1 + *N. sativa* 16%. Different superscript letters in each column/week indicate significant (*p* < 0.05) differences between groups, and NS stands for not significant (*p* > 0.05).

**Table 6:** Effects of *N. sativa* on biochemical parameters (Mean ± SEM) of broilers.

Age (day)	Group	Chol (mg/dL)	Trigl (mg/dL)	ALP (U/L)	ALT (U/L)	AST (U/L)
Day1	T0–T7	304.9 ± 6.7NS	179.825 ± 5.1NS	12.03 ± 0.17NS	7.9 ± 0.3NS	218.4 ± 2.6NS
Day 7	T0	154.8 ± 2.7a	137.0 ± 2.7a	10.38 ± 0.12a	9.2 ± 0.3a	241.2 ± 2.3a
	T1	155.2 ± 1.9a	132.0 ± 1.8b	10.28 ± 0.22a	9.6 ± 0.4a	246.8 ± 2.6a
	T2	156.2 ± 1.6a	115.8 ± 2.0c	10.03 ± 0.13a	9.8 ± 0.3a	237.6 ± 2.4b
	T3	157.2 ± 1.5a	115.4 ± 2.1c	9.94 ± 0.10a	10.6 ± 0.4b	236.8 ± 2.5b
	T4	155.4 ± 1.2a	112.4 ± 2.1c	9.92 ± 0.10a	11.2 ± 0.5b	233.8 ± 2.8bc
	T5	155.8 ± 1.5a	107.2 ± 1.9d	9.91 ± 0.13a	11.0 ± 0.3b	228.4 ± 2.4cd
	T6	154.0 ± 1.7a	105.8 ± 2.0d	9.91 ± 0.10a	9.2 ± 0.3a	227.6 ± 2.6d
	T7	154.6 ± 1.3a	103.4 ± 2.4d	9.82 ± 0.10a	8.0 ± 0.4c	227.0 ± 2.2d
Day 14	T0	154.4 ± 1.3a	173.2 ± 3.4a	8.96 ± 0.14a	9.7 ± 0.4ac	234.6 ± 2.4a
	T1	150.4 ± 2.4a	172.0 ± 2.7a	8.98 ± 0.20a	9.6 ± 0.3ac	234.0 ± 2.5a
	T2	151.6 ± 1.7a	167.4 ± 3.1b	8.78 ± 0.10ab	10.0 ± 0.5a	230.0 ± 2.5a
	T3	150.4 ± 1.5a	166.2 ± 2.9b	8.58 ± 0.10ab	10.0 ± 0.5a	224.2 ± 2.8b
	T4	153.2 ± 3.1a	161.2 ± 3.5c	8.74 ± 0.10ab	11.6 ± 0.6b	194.8 ± 2.9c
	T5	153.4 ± 1.9a	157.6 ± 3.7c	8.58 ± 0.16ab	9.8 ± 0.5a	181.6 ± 2.6d
	T6	154.8 ± 2.5a	151.8 ± 3.1c	8.50 ± 0.10ab	9.6 ± 0.4ac	178.4 ± 2.6d
	T7	151.0 ± 3.1a	145.0 ± 3.7d	8.42 ± 0.11b	9.2 ± 0.7c	149.4 ± 2.3e
Day 21	T0	167.4 ± 2.3a	163.2 ± 3.4a	6.98 ± 0.14ab	10.0 ± 0.4ab	215.6 ± 2.3a
	T1	165.6 ± 3.3a	165.0 ± 2.7a	7.02 ± 0.20a	10.6 ± 0.3a	215.2 ± 2.5a
	T2	163.4 ± 1.5abc	164.4 ± 4.2a	6.82 ± 0.10abc	11.0 ± 0.7a	206.6 ± 2.3ab
	T3	161.4 ± 2.9abc	160.4 ± 2.9a	6.78 ± 0.10abc	11.2 ± 0.5a	200.2 ± 2.2 b
	T4	160.8 ± 1.9abc	160.2 ± 4.5ab	6.60 ± 0.18abc	11.2 ± 0.6a	199.4 ± 2.3 b
	T5	160.6 ± 1.9abc	158.0 ± 4.7 b	6.58 ± 0.10abc	9.8 ± 0.5 b	190.4 ± 2.9bc
	T6	158.8 ± 2.5bc	151.6 ± 5.1c	6.54 ± 0.10bc	9.6 ± 0.4b	178.0 ± 2.2c
	T7	157.0 ± 2.4c	144.4 ± 3.7d	6.44 ± 0.10c	8.2 ± 0.7c	148.8 ± 2.6d
Day 28	T0	156.4 ± 2.2a	159.2 ± 3.7a	6.34 ± 0.13a	10.2 ± 0.2a	191.6 ± 2.2a
	T1	154.4 ± 2.3a	159.4 ± 2.7a	6.38 ± 0.20a	10.2 ± 0.2a	194.0 ± 2.3a
	T2	154.4 ± 2.1a	156.4 ± 3.4ab	6.18 ± 0.10ab	10.8 ± 0.2a	184.0 ± 2.3b
	T3	154.2 ± 1.5a	152.8 ± 3.1b	6.12 ± 0.10ab	11.0 ± 0.1b	181.2 ± 2.8b
	T4	154.4 ± 2.9a	147.4 ± 2.9b	5.98 ± 0.10abc	11.6 ± 0.2b	175.8 ± 2.9cb
	T5	148.6 ± 2.5b	138.4 ± 3.5c	5.92 ± 0.15abc	10.4 ± 0.2bac	170.6 ± 2.8c
	T6	148.4 ± 2.4b	131.6 ± 3.7d	5.88 ± 0.08bc	10.0 ± 0.2bac	164.4 ± 2.8 d
	T7	144.0 ± 2.4b	128.0 ± 4.1d	5.66 ± 0.09c	9.80 ± 0.1c	138.4 ± 2.2e
Day 35	T0	150.4 ± 2.3a	153.2 ± 3.7a	5.54 ± 0.13a	10.6 ± 0.1a	182.6 ± 2.4a
	T1	149.4 ± 3.3a	150.0 ± 3.4ab	5.58 ± 0.20a	10.2 ± 0.2a	182.2 ± 2.2a
	T2	149.6 ± 3.1a	147.8 ± 2.7b	5.38 ± 0.08ab	10.2 ± 0.2a	171.0 ± 2.8 b
	T3	145.4 ± 2.9ab	142.8 ± 2.1bc	5.32 ± 0.10ab	10.4 ± 0.1a	165.6 ± 2.8bc
	T4	145.8 ± 2.5ab	138.4 ± 2.9 cd	5.18 ± 0.09abc	11.8 ± 0.2b	162.8 ± 2.5cd
	T5	144.8 ± 2.4ab	133.0 ± 2.5de	5.12 ± 0.15abc	10.0 ± 0.2a	157.4 ± 2.7d
	T6	139.8 ± 2.2bc	130.2 ± 2.7e	5.08 ± 0.08bc	10.0 ± 0.1a	141.4 ± 2.7e
	T7	136.0 ± 2.3c	126.2 ± 2.1	4.84 ± 0.08c	9.0 ± 0.1c	133.4 ± 2.0e
Day 42	T0	136.4 ± 2.1a	132.2 ± 3.4a	5.14 ± 0.13a	10.8 ± 0.1a	144.6 ± 2.5a
	T1	135.4 ± 1.5a	132.0 ± 2.7a	5.16 ± 0.20a	10.6 ± 0.2a	144.2 ± 2.4a
	T2	133.4 ± 1.9a	127.2 ± 2.1ab	4.98 ± 0.06ab	10.6 ± 0.2a	133.0 ± 2.4 b
	T3	130.8 ± 1.9a	124.4 ± 2.9 bc	4.92 ± 0.10ab	10.8 ± 0.2a	127.6 ± 2.8bc
	T4	129.6 ± 2.3a	123.4 ± 2.5bc	4.74 ± 0.09abc	12.0 ± 0.1b	124.8 ± 2.9c
	T5	121.2 ± 2.4b	121.6 ± 2.7c	4.72 ± 0.15abc	10.0 ± 0.3c	119.4 ± 2.8c
	T6	117.8 ± 1.5b	114.4 ± 2.1de	:	9.4 ± 0.2c	103.4 ± 2.9d
	T7	108.0 ± 2.9c	110.6 ± 3.7e	4.48 ± 0.08c	8.8 ± 0.2d	95.4 ± 2.2d

*Note*. Chol: cholesterol, Trigl: triglyceride, ALP: alkaline phosphatase, ALT: alanine transaminase, and AST: aspartate transaminase. T0 = control 1 (without vaccination and without supplementation of *N. sativa* in diet), T1 = control 2 (T0 + vaccination), T2 = T1 + *N. sativa* 0.5%, T3 = T1 + *N. sativa* 1%, T4 = T1 + *N. sativa* 2%, T5 = T1 + *N. sativa* 4%, T6 = T1 + *N. sativa* 8%, and T7 = T1+*N. sativa* 16%. Different superscript letters in each column/week indicate significant (*p* < 0.05) differences between groups, and NS stands for not significant (*p* > 0.05).

## Data Availability

Data used to support the findings of this study are included within the article.

## Abbreviations

N: *Nigella*
V: Vaccinated
RBC: Red blood cells
PCV: Packed cell volume
MCV: Mean corpuscular volume
MCH: Mean corpuscular hemoglobin
MCHC: Mean corpuscular hemoglobin concentration
WBC: White blood cells.
